# SIRT1-mediated epigenetic downregulation of plasminogen activator inhibitor-1 prevents vascular endothelial replicative senescence

**DOI:** 10.1111/acel.12247

**Published:** 2014-07-18

**Authors:** Yan-Zhen Wan, Peng Gao, Shuang Zhou, Zhu-Qin Zhang, De-Long Hao, Li-Shan Lian, Yong-Jun Li, Hou-Zao Chen, De-Pei Liu

**Affiliations:** 1 State Key Laboratory of Medical Molecular Biology, Institute of Basic Medical Sciences, Chinese Academy of Medical Sciences & Peking Union Medical CollegeBeijing, China; 2 Department of Vascular Surgery, Peking Union Medical College Hospital, Peking Union Medical College, Chinese Academy of Medical ScienceBeijing, 100730, China

**Keywords:** atherosclerosis, endothelial replicative senescence, epigenetic, PAI-1, H4K16 acetylation, SIRT1

## Abstract

The inactivation of plasminogen activator inhibitor-1 (PAI-1) has been shown to exert beneficial effects in age-related vascular diseases. Limited information is available on the molecular mechanisms regarding the negatively regulated expression of PAI-1 in the vascular system. In this study, we observed an inverse correlation between SIRT1, a class III histone deacetylase, and PAI-1 expression in human atherosclerotic plaques and the aortas of old mice, suggesting that internal negative regulation exists between SIRT1 and PAI-1. SIRT1 overexpression reversed the increased PAI-1 expression in senescent human umbilical vein endothelial cells (HUVECs) and aortas of old mice, accompanied by decreased SA-β-gal activity *in vitro* and improved endothelial function and reduced arterial stiffness *in vivo*. Moreover, the SIRT1-mediated inhibition of PAI-1 expression exerted an antisenescence effect in HUVECs. Furthermore, we demonstrated that SIRT1 is able to bind to the PAI-1 promoter, resulting in a decrease in the acetylation of histone H4 lysine 16 (H4K16) on the PAI-1 promoter region. Thus, our findings suggest that the SIRT1-mediated epigenetic inhibition of PAI-1 expression exerts a protective effect in vascular endothelial senescence.

## Introduction

Epidemiological and autopsy studies have shown that aging is a major risk factor for cardiovascular diseases (Lakatta & Levy, 2003). Aging is accompanied by changes in vascular function, such as increased stiffness and reduced compliance, which contribute to the increased incidence of clinical diseases and predict the future risk of developing cardiovascular diseases, such as atherosclerosis in the elderly (Lakatta, 2003). Endothelial senescence represents one of the major characteristics of vascular aging and has been implicated in the development of atherosclerosis (Minamino *et al*., 2002; Minamino & Komuro, 2007; Bai *et al*., 2012). Senescent endothelial cells might contribute to vascular aging and atherosclerosis through alterations in gene expression in these cells. Therefore, the elucidation of the molecular mechanisms involved in the regulation of vascular cell senescence could provide information for the development of drugs to manipulate and normalize gene expression in vascular aging and atherosclerosis.

Plasminogen activator inhibitor-1 (PAI-1), one of the important key genes expressed in endothelial cells, is the most important serine protease inhibitor of tissue-type (t-PA) and urokinase-type (u-PA) plasminogen activators in plasma (Van De Craen *et al*., 2012). Accumulating evidence has shown that increase in plasma PAI-1 levels is an independent risk factor for cardiovascular diseases. Increased levels of PAI-1 are associated with AS and thrombosis resulting from impaired fibrinolysis (Schneiderman *et al*., 1992; Yamamoto *et al*., 2002; Cesari *et al*., 2010), and age-related increase of PAI-1 can also be used as a marker of endothelial senescence (Comi *et al*., 1995). Moreover, the PAI-1 knockout *in vivo* protects against several age-related diseases, such as atherosclerosis, hypertension, and stroke (Eitzman *et al*., 2000; Kaikita *et al*., 2001; Tjarnlund-Wolf *et al*., 2012), which closely associated with vascular aging. Although PAI-1 is sufficient for the induction of replicative senescence (Kortlever *et al*., 2006) and has been implicated in vascular aging and age-related vascular diseases, limited information is available on the molecular mechanisms underlying the negatively regulated expression of PAI-1 in the vascular system.

Calorie restriction extends the lifespan of yeast, worms, flies, and mammals and decreases the incidence of age-associated disorders such as cardiovascular diseases and diabetes in rhesus monkeys (Colman *et al*., 2009; Guarente, 2012). The beneficial effects of low caloric intake are mediated by silent information regulator 2 (Sir2) proteins, or sirtuins, a conserved nicotinamide adenine dinucleotide-dependent protein deacetylase that plays critical roles in aging and age-associated diseases (Finkel *et al*., 2009; Haigis & Sinclair, 2010). SIRT1, the best characterized mammalian sirtuin, is highly expressed in the vasculature (Potente *et al*., 2007). Previous studies have shown that SIRT1 plays a critical role in the endothelial-dependent control of the vascular tone (Mattagajasingh *et al*., 2007; Zhang *et al*., 2008; Zhou *et al*., 2011) and a protective role in atherosclerotic vascular diseases (Zhang *et al*., 2008; Stein *et al*., 2010; Gorenne *et al*., 2013). Moreover, SIRT1 overexpression or activation in endothelial cells displays protective effects on oxidative stress-induced premature senescence (Ota *et al*., 2007, 2008; Kao *et al*., 2010; Zu *et al*., 2010; Chen *et al*., 2012). However, it remains unclear whether SIRT1 prevents replicative vascular senescence and by which SIRT1 facilitates the effects of antireplicative senescence.

In the present study, we detected an internal inverse correlation between SIRT1 and PAI-1 expression in human atherosclerotic plaques and the aortas of aged mice. SIRT1 overexpression reversed the increased PAI-1 expression in senescent endothelial cells and aortas of old mice, accompanied by improved endothelial function and reduced arterial stiffness. Moreover, we demonstrated that SIRT1 protected against replicative senescence in endothelial cells through a novel mechanism involving the epigenetic downregulation of PAI-1 expression.

## Results

### The expression of SIRT1 and PAI-1 shows inverse correlation in human atherosclerosis plaques

**Figure 1:**
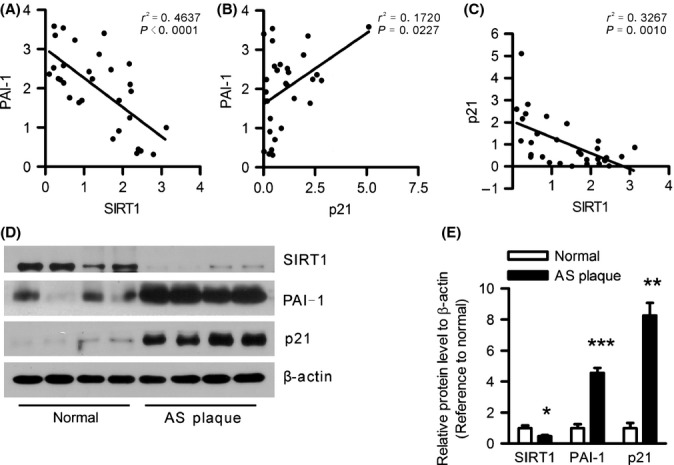
SIRT1 is inversely correlated with PAI-1 in human atherosclerotic (AS) plaques. The relative gene expression was calculated using the densitometric values of immunoblots for PAI-1, SIRT1, and p21 proteins divided by β-actin as an internal reference. Pearson’s correlation coefficient (r) and statistical significance values (p) are shown. (A) The correlation analysis between SIRT1 and PAI-1 protein levels in 30 AS plaques is displayed using a XY scatter plot, and every plot represents a single sample collected from a patient. (B) Correlation between PAI-1 levels and p21 in human AS lesions. (C) Correlation between SIRT1 levels and p21 in human AS lesions. (D) Representative immunoblots for SIRT1, PAI-1, p21, and β-actin in the carotid arteries of normal control and human AS lesions obtained from four independent individuals. (E) The bar graphs show the results of the densitometric analysis of the immunoblots for SIRT1, PAI-1, and p21 protein. The densitometric quantification was normalized to β-actin. Data are presented as the mean±SEM of the SIRT1/β-actin, PAI-1/β-actin, and p21/β-actin expression ratios (*n* = 4 for normal control and *n* = 30 for human AS lesions). *P < 0.05, **P < 0.01 and ***P < 0.001 vs. the carotid arteries of the normal control.

In a previous study, we showed that SIRT1 overexpression in the endothelium protects against atherogenesis (Zhang *et al*., 2008). Considering that increased levels of plasma PAI-1 might be an important risk factor for atherosclerosis, we hypothesized that SIRT1 is inversely correlated with PAI-1 expression in atherosclerosis. To confirm this hypothesis, we collected 30 samples of carotid arteries from patients who underwent carotid endarterectomy for symptomatic disease and examined the expression patterns of SIRT1 and PAI-1 in human atherosclerotic plaques. As shown in Fig. 1, the PAI-1 protein levels showed a strong negative correlation with SIRT1 (Figs 1A and S1) and exhibited a positive correlation with the established senescence-associated marker p21 (Figs 1B and S1), while SIRT1 also showed a relatively weak negative correlation with p21 (Figs 1C and S1). Next, we examined the expression of SIRT1 and PAI-1 in normal carotid arteries and atherosclerotic plaques from human donors and observed a lower SIRT1 level accompanied by the upregulation of PAI-1 and increased p21 protein expression in human atherosclerotic plaques compared with the control group (Fig. 1D,E). These data suggest that SIRT1 might negatively regulate PAI-1 expression in atherosclerotic vascular diseases.

### SIRT1 expression is negatively correlated with PAI-1 level in senescent HUVECs and aortas of aged mice

**Figure 2:**
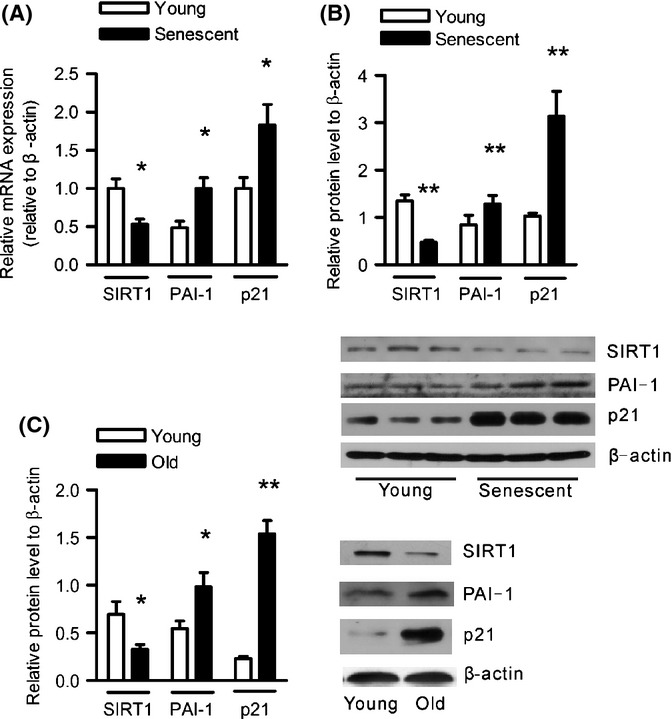
SIRT1 expression is negatively correlated with PAI-1 level *in vitro* and *in vivo* in vascular aging. Young (PDL6-8) and senescent (PDL41-44) HUVECs were used to analyze the mRNA (A) and protein levels (B) of SIRT1, PAI-1, and p21. SIRT1, PAI-1, and p21 mRNAs were analyzed using real-time polymerase chain reaction (PCR), and the proteins were analyzed using Western blotting with anti-SIRT1, anti-PAI-1, and anti-p21 antibodies. The RNA level was normalized to the internal control β-actin and expressed relative to young HUVECs. The densitometric quantification of immunoblots for SIRT1, PAI-1, and p21 proteins was normalized to β-actin. Data are shown as the mean±SEM of three independent samples. *P < 0.05 and **P < 0.01 vs. young cells. (C) Western blotting analysis of SIRT1, PAI-1, and p21 protein levels was performed using the aortas of young (2–3 month old) and old (18–22 month old) mice. The densitometric quantification was normalized to β-actin, and data are shown as the mean ± SEM for six mice. *P < 0.05 and **P < 0.01 vs. young mice.

To further investigate the internal correlation between SIRT1 expression and the PAI-1 level in vascular aging, we first compared the expression levels of the two molecules in the continuous passage of HUVECs *in vitro*. PDL41-44 HUVECs showed a flattened and enlarged cell morphology and expressed much more SA-β-gal activity (Fig. S2A). No apoptosis signal was observed using Hoechest staining (Fig. S2B). Senescent HUVECs entered into an irreversible growth arrest status, verified by the markedly increased expression of senescence-associated molecules, such as p16, compared with PDL6-8 young HUVECs (Fig. S3A). In addition, a lower SIRT1 level was observed accompanied by the upregulation of PAI-1 and increased expression of p21 in senescent HUVECs at both the mRNA and protein levels (Fig. 2A,B). To further confirm this phenomenon *in vivo*, we collected aorta tissues from 2- to 3-month-old (young) and 20- to 22-month-old (old) C57 mice. Consistently, SIRT1 expression was significantly decreased, while PAI-1 and p21 protein levels were enhanced in the aortas of old mice (Fig. 2C), and the plasma levels of active PAI-1 were drastically increased in old mice (Fig. S3B). These results suggest internal negative regulation between SIRT1 and PAI-1 expression in vascular aging.

### SIRT1 protects against endothelial replicative senescence and inhibits PAI-1 expression in HUVECs

**Figure 3:**
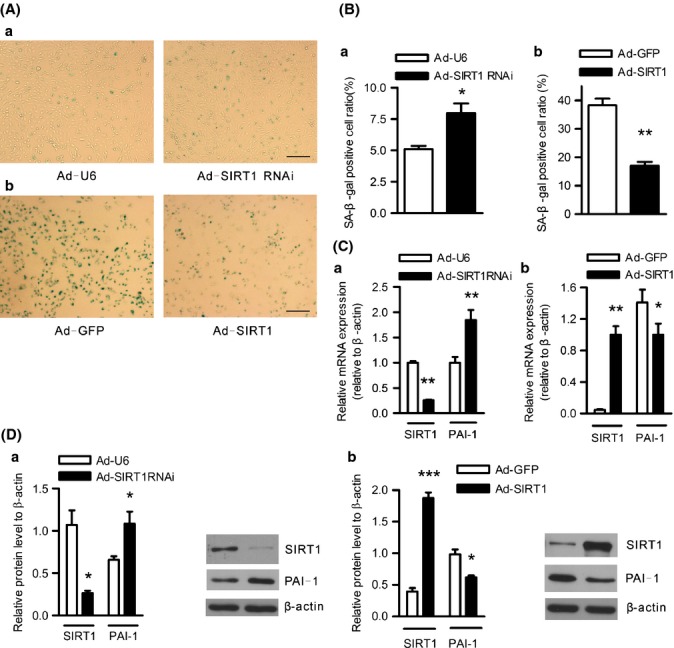
SIRT1 knockdown induces senescence and PAI-1 expression in young HUVECs, while SIRT1 overexpression protects against senescence and inhibits PAI-1 expression in senescent HUVECs. (A) Senescence was evaluated through the SA-β-gal staining of young HUVECs infected with Ad-U6 or Ad-SIRT1 RNAi (a) and senescent HUVECs infected with Ad-GFP or Ad-SIRT1 (b) cultured for an additional 72 h. Blue-stained cells were considered senescent. The bar represents 200 μm. (B) The percentage of SA-β-gal-positive cells on young HUVECs (a) and senescent HUVECs (b) were quantified using commercial software. Data are presented as the mean±SEM of three independent experiments. *P < 0.05 vs. control Ad-U6. **P < 0.01 vs. control Ad-GFP. (C–D) Young HUVECs were infected with Ad-U6 or Ad-SIRT1 RNAi, (a) while senescent HUVECs were infected with Ad-GFP or Ad-SIRT1 (b), followed by culturing for an additional 48 h. SIRT1 and PAI-1 mRNA (C) and protein (D) levels were analyzed using real-time RT–PCR and Western blotting, respectively. The RNA and protein levels were normalized to the internal control β-actin. Data are presented as the mean±SEM of three independent experiments. *P < 0.05 vs. corresponding control Ad-U6 or Ad-GFP. **P < 0.01 vs. corresponding control Ad-U6 or Ad-GFP. ***P < 0.001 vs. Ad-GFP.

To investigate the role of SIRT1 activity in the regulation of PAI-1 expression, we treated 293A cells with three different sirtuin inhibitors (sirtinol, nicotinamide, and suramin) and analyzed PAI-1 expression. The sirtuin inhibitors significantly increased PAI-1 mRNA (Fig. S4A). Next, we treated HUVECs with sirtinol or another SIRT1-specific inhibitor, EX527. Results showed that both of the inhibitors induced PAI-1 expression in young HUVECs (Fig. S4B,C). Conversely, treatment of senescent HUVECs with SRT1720, a SIRT1-specific activator, significantly inhibited PAI-1 expression at both the mRNA and protein levels (Fig. S4D–F). Previous studies have shown that SIRT1 inhibition induces endothelial cell senescence (Ota *et al*., 2007; Zhou *et al*., 2011). Consistently, we observed that the adenovirus-mediated SIRT1 knockdown directly induced senescence in young HUVECs (Fig. 3A-a,B-a). Conversely, SIRT1 overexpression significantly decreased SA-β-gal activity in senescent HUVECs (Fig. 3A-b,B-b), suggesting that SIRT1 protects against endothelial replicative senescence. Correspondingly, PAI-1 expression was dramatically enhanced after SIRT1 knockdown at both the mRNA and protein levels in young HUVECs (Fig. 3C-a,D-a), while SIRT1 overexpression markedly inhibited PAI-1 expression at both the mRNA and protein levels in senescent HUVECs (Fig. 3C-b, D-b). These findings suggest that the reduction of SIRT1 might induce PAI-1 overexpression in senescent endothelial cells.

### Endothelium-specific SIRT1 overexpression reverses PAI-1 upregulation and protects against age-dependent vascular dysfunction and arterial stiffness in aged mice

**Figure 4:**
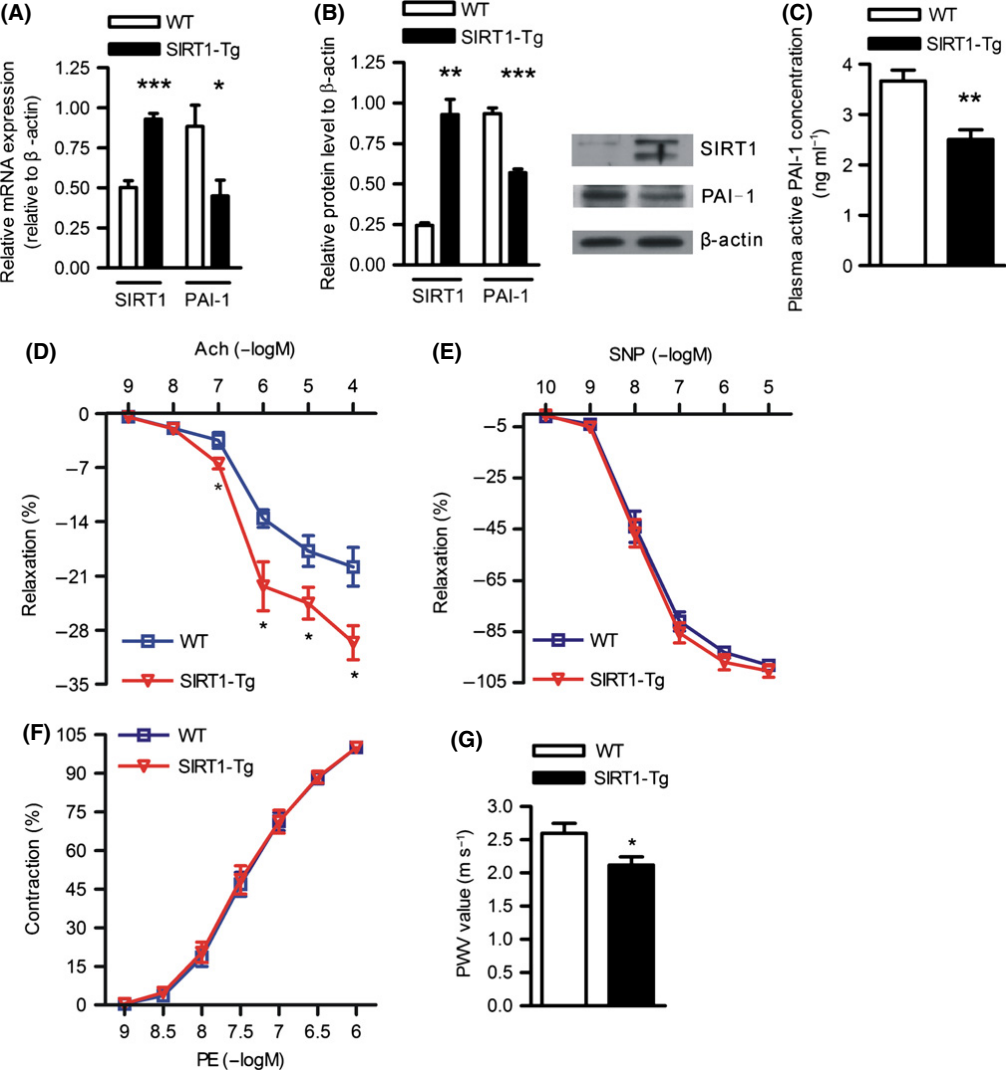
SIRT1 overexpression in the endothelium shows decreased PAI-1 expression and improved age-dependent vascular dysfunction and arterial stiffness. SIRT1 and PAI-1 mRNA (A) and protein (B) expressions in the aortas of old WT and SIRT1-Tg mice. The bar graphs show the relative quantitation of SIRT1 and PAI-1 mRNA or densitometric analysis of SIRT1 and PAI-1 protein immunoblots. Data are presented as the mean±SEM (*n* = 6 in each group). *P < 0.05, **P < 0.01, and ***P < 0.001 vs. old WT mice. Immunoblots for SIRT1, PAI-1, and β-actin are representatives of six independent experiments. (C) The active PAI-1 protein concentration in plasma of WT and SIRT1-Tg old mice. *n* = 16 for WT old mice and *n* = 10 for SIRT1-Tg old mice. **P < 0.01 vs. old WT mice. (D-F) Introduction of SIRT1 in the endothelium significantly conserves the endothelium-dependent vasorelaxation in old mice. Isometric tension studies in aortic rings from two groups of mice (*n* = 9 for old WT mice and *n* = 11 for old SIRT1-Tg mice). (D) WT old mice exhibited impaired endothelium-dependent relaxation in response to Ach compared with SIRT1-Tg old mice. (E) Vessel relaxation in response to NO-donor SNP, the endothelium-independent agonist, was identical in the two groups. (F) Vessel contractions mediated through PE were identical in WT and SIRT1-Tg old mice. Data are presented as the mean ± SEM; *P < 0.05 vs. WT old mice. G: SIRT1-Tg old mice show decreased arterial stiffness. The data collected from PWV assay in LCCAs of WT, and SIRT1-Tg old mice were analyzed using commercial software. *n* = 16 for WT old mice and *n* = 10 for SIRT1-Tg old mice. Data are presented as the mean±SEM. *P < 0.05 vs. WT old mice.

To further examine whether SIRT1 inhibits increased PAI-1 expression in vascular aging, we used endothelium-specific SIRT1-Tg old mice to examine whether the introduction of SIRT1 regulates PAI-1 expression *in vivo*. SIRT1 overexpression was confirmed in the aortas of endothelium-specific SIRT1-Tg old mice at both the mRNA and protein levels (Fig. 4A,B). SIRT1-Tg old mice displayed dramatically decreased mRNA and protein levels of aging-related molecules, such as p53 and p21, compared with their age-matched WT controls (Fig. S5). Importantly, PAI-1 mRNA and protein were significantly decreased in the aortas of SIRT1-Tg old mice compared with those of WT mice (Fig. 4A,B). In addition, we assayed PAI-1 activity in mouse plasma and observed that the plasma collected from SIRT1-Tg old mice contained much less active PAI-1 molecules compared with those of WT mice (Fig. 4C). These results indicate that SIRT1 overexpression reverses the increased PAI-1 expression in aged mice. Furthermore, we detected PAI-1 expression in the aortas of old SIRT1(+/+) and SIRT1(+/−) mice and found that SIRT1(+/−) old mice displayed increased PAI-1 protein level compared with their age-matched WT controls, suggesting that SIRT1 downregulation promotes PAI-1 expression in aged mice (Fig. S6). As increased stiffness and reduced compliance are major characteristics of aged arteries, increased PAI-1 level has been considered as a hallmark of endothelial dysfunction (Brodsky *et al*., 2002) and positively associated with vascular stiffness (Lieb *et al*., 2009). To examine whether the suppression of PAI-1 expression and activity through SIRT1 overexpression in the endothelium affects vascular function, we performed isometric tension studies to evaluate endothelium-dependent vasorelaxation in mice. Endothelium-dependent relaxation to acetylcholine (Ach) was significantly improved in SIRT1-Tg old mice compared with their age-matched controls (Fig. 4D). No difference was observed in vascular dilations in response to sodium nitroprusside (SNP) or contractions in response to phenylephrine (PE) between aortas obtained from old SIRT1-Tg and WT mice (Fig. 4E,F). These results indicate that overexpression of SIRT1 improves endothelial function in the aortas of aged mice *in vivo*. As stiffness of the carotid arteries is most sensitive to vascular aging and easily achieved in a PWV assay using the ultrasonic detection method, the left common carotid arteries (LCCA) of mice were selected to detect vascular stiffness *in vivo* (Williams *et al*., 2007). As expected, old SIRT1-Tg mice showed decreased PWV compared with WT controls, suggesting that SIRT1 overexpression improves age-dependent vascular stiffness (Fig. 4G).

### The anti-aging effect of SIRT1 is mediated by inhibition of PAI-1 expression

**Figure 5:**
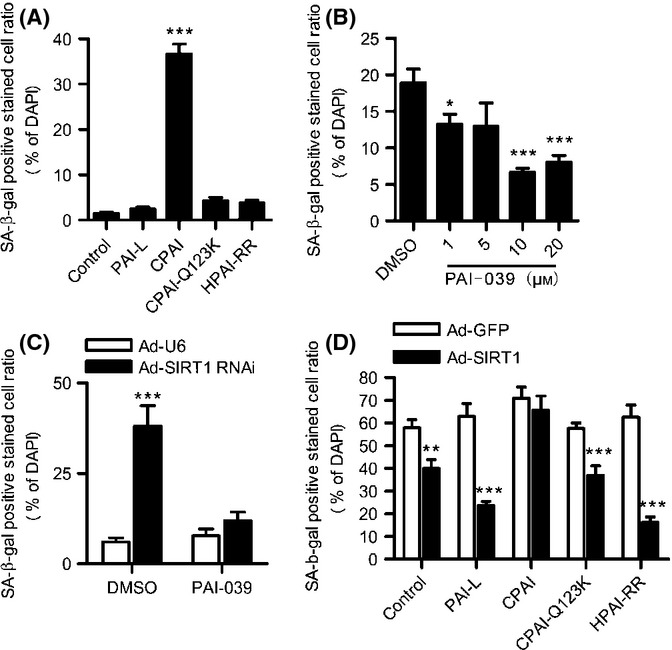
SIRT1 protects against endothelial senescence through the downregulation of PAI-1 in HUVECs. (A) Young HUVECs were cultured for 72 h in medium containing 25 nm PAI-L, CPAI, CPAI-Q123K, or HPAI-RR, and subsequently, SA-β-gal staining assay was performed. The bar graphs show the quantitative analysis of the SA-β-gal-positive cell ratio normalized to DAPI-stained cell number. Data are shown as the mean ± SEM of three independent experiments. ***P < 0.001 vs. PAI-L. (B) Senescent HUVECs were cultured for 72 h in medium containing 1, 5, 10, or 20 μM of PAI-039, and subsequently, the SA-β-gal-positive cell ratio was calculated. Data are shown as the mean±SEM of three independent experiments. *P < 0.05 and ***P < 0.001 vs. DMSO. (C) Young HUVECs were infected with Ad-U6 or Ad-SIRT1 RNAi for 12 h and cultured in medium containing 10 μM PAI-039 for an additional 72 h. Subsequently, the SA-β-gal-positive cell ratio was calculated. Data are shown as the mean±SEM of three independent experiments. ***P < 0.001 vs. Ad-U6. (D) Senescent HUVECs were infected with Ad-GFP or Ad-SIRT1 for 12 h and cultured in medium containing 25 nM PAI-L, CPAI, CPAI-Q123K, or HPAI-RR for an additional 72 h. Subsequently, the SA-β-gal-positive cell ratio was calculated. Data are shown as the mean±SEM of three independent experiments. **P < 0.01 and ***P < 0.001 vs. Ad-GFP.

To investigate the role of PAI-1 in the anti-aging effect of SIRT1 in HUVECs, we first examined the effectiveness of recombinant human PAI-1 proteins in young HUVECs, including CPAI (stable mutant form), CPAI-Q123K (stable vitronectin reduced binding mutant), and HPAI-RR (substrate double mutant form, with no detectable antiproteolytic activity), while PAI-L (wild-type latent fraction) was used as a negative control. CPAI-Q123K, HPAI-RR, and the negative control PAI-L all failed to directly induce HUVEC senescence, whereas CPAI resulted in SA-β-gal-positive staining in nearly 40% of HUVECs (Fig. 5A). Moreover, treatment of senescent HUVECs with the PAI-1 inhibitor PAI-039 significantly decreased the senescent cell ratio in a dose-dependent manner (Fig. 5B). These results indicate that PAI-1 is sufficient to induce the senescence of HUVECs, and its binding to vitronectin and antiproteolytic activity are indispensable for the pro-senescence effect of PAI-1. To examine whether endothelial senescence induced by the reduction of SIRT1 could be blocked through the inhibition of PAI-1, we added PAI-039 into young HUVECs infected with Ad-U6 or Ad-SIRT1 RNAi and observed that PAI-1 inhibition significantly reversed the senescence induced by SIRT1 knockdown in HUVECs (Fig. 5C). Next, we treated senescent HUVECs infected with Ad-GFP or Ad-SIRT1 with the four types of PAI-1 protein to examine whether PAI-1 participated in the antisenescence effect of SIRT1. The results showed that treatment with an exogenous stable form of PAI-1 CPAI, but not CPAI-Q123K, HPAI-RR, and PAI-L, nearly completely blocked the antisenescence effect of adenovirus-mediated SIRT1 overexpression in senescent HUVECs (Fig. 5D), suggesting that SIRT1-mediated inhibition of PAI-1 expression contributes to the anti-aging effect of this protein in HUVECs.

### SIRT1 negatively regulates PAI-1 expression through epigenetic chromatin modification

As SIRT1 regulates PAI-1 expression at the transcriptional level, we performed a PAI-1 promoter luciferase assay in 293A cells to further investigate the detailed mechanism of PAI-1 regulation by SIRT1. Several polymorphisms have been identified in the promoter of PAI-1 gene, among which the common PAI-1–675(4G/5G) (rs1799889) and PAI-1–844A/G (rs2227631) polymorphisms have been most extensively studied. The haplotype containing the -675 4G and the –844 A alleles was associated with higher transcriptional activity compared with the -675 5G and –844G-containing haplotype (Hultman *et al*., 2010). Therefore, a series of human PAI-1 promoter luciferase reporter plasmids harboring different polymorphisms were constructed, including -675 4G/-844A, -675 4G/-844G, -675 5G/-844A, and -675 5G/-844G. Consistent with previous reports, the results of luciferase activity assay demonstrated that compared with the lowest activity of promoter containing -675 5G/-844G polymorphism, the -675 4G/-844A polymorphism possessed the highest activity. However, neither basic nor p53-activated PAI-1 promoter activity was affected by SIRT1 overexpression (Fig. S7A). Similarly, SIRT1 inhibition through EX527 treatment did not obviously affect the activity of all four types of PAI-1 promoters (Fig. S7B), suggesting that SIRT1 did not directly affect PAI-1 promoter activity.

**Figure 6:**
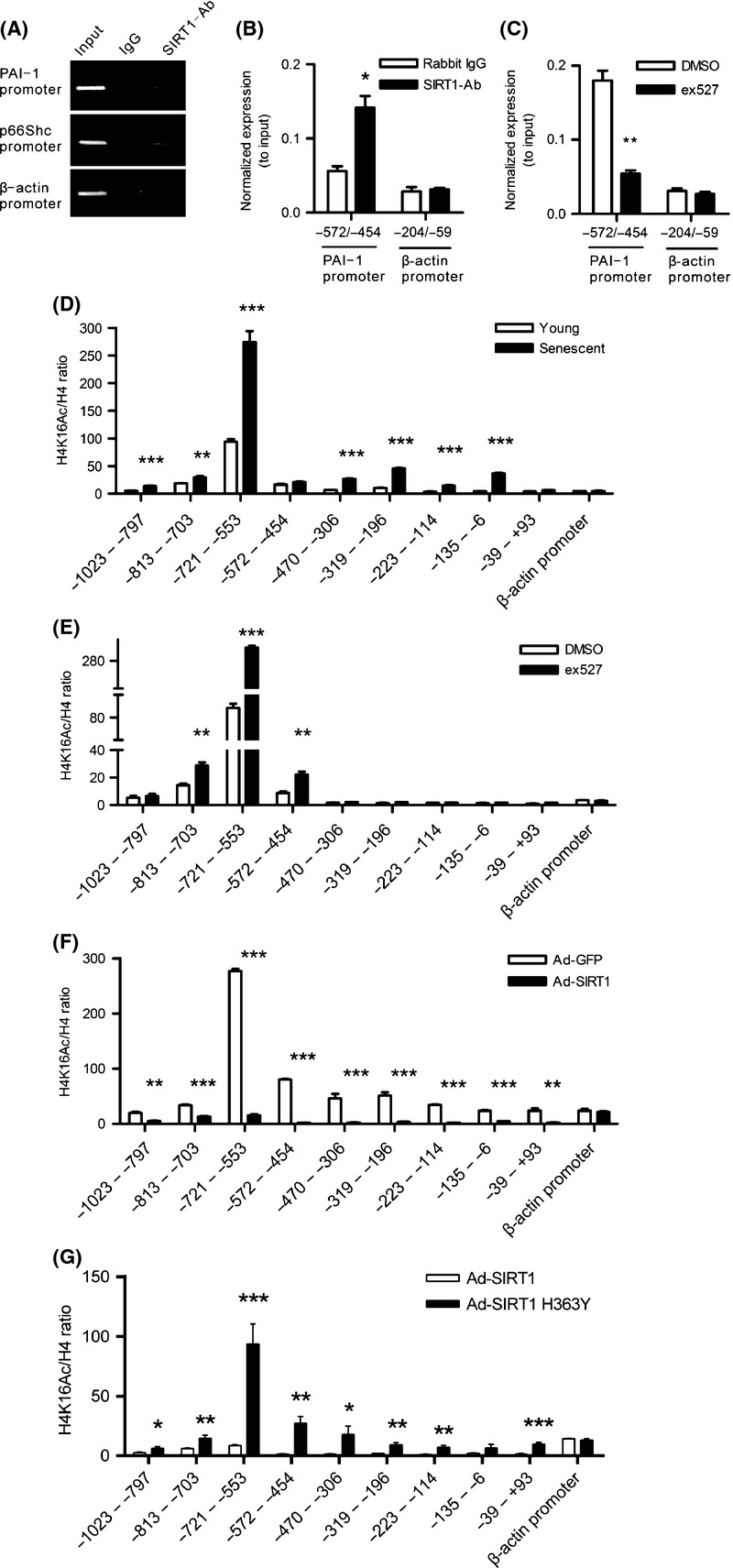
SIRT1 negatively regulates PAI-1 expression through epigenetic chromatin modification. (A–B) ChIP assays were performed with chromatin prepared from HUVECs. The chromatin was immunoprecipitated with normal rabbit IgG or an antibody against SIRT1. (A) Precipitated genomic DNA was analyzed through semiquantitative PCR using primers for the specific PAI-1 promoter region (−572 to −454 bp). The p66Shc promoter (−508 to −250 bp) was used as a positive control, and the β-actin promoter (−204 to −59 bp) was used as a negative control. (B) Precipitated genomic DNA was analyzed through real-time PCR using primers for the specific PAI-1 promoter region (−572 to −454 bp), and the β-actin promoter was used as a negative control. Data are shown as the mean±SEM of three independent experiments. *P < 0.05 vs. rabbit IgG. (C) ChIP assays were performed on HUVECs treated with 1 μm EX527 for 6 h. The chromatin was immunoprecipitated with normal rabbit IgG or an antibody against SIRT1. Precipitated genomic DNA was analyzed through real-time PCR using primers for the specific PAI-1 promoter region (−572 to −454 bp). The β-actin promoter (−204 to −59 bp) was used as a negative control. Data are shown as the mean±SEM of three independent experiments. **P < 0.01 vs. DMSO. (D) ChIP assays were performed with chromatin prepared from young and senescent HUVECs. The chromatin was immunoprecipitated with normal rabbit IgG or antibodies against H4K16Ac and H4, and precipitated genomic DNA was analyzed through real-time PCR using different primers for the different regions of PAI-1 promoter. The β-actin promoter (−204 to −59 bp) was used as a negative control. Data are shown as the mean±SEM of three independent experiments. **P < 0.01 and ***P < 0.001 vs. young HUVECs. (E) ChIP assays were performed on HUVECs treated with 1 μM EX527 for 6 h. The chromatin was immunoprecipitated with normal rabbit IgG or antibodies against H4K16Ac and H4. Precipitated genomic DNA was analyzed through real-time PCR using different primers for the different regions of PAI-1 promoter. The β-actin promoter (−204 to −59 bp) was used as a negative control. Data are shown as the mean±SEM of three independent experiments. **P < 0.01 and ***P < 0.001 vs. DMSO. (F) The acetylation of histone H4K16 on the PAI-1 promoter was dramatically decreased by SIRT1 overexpression in senescent HUVECs. Senescent HUVECs were infected with Ad-GFP or Ad-SIRT1 and cultured for an additional 48 h. The chromatin was immunoprecipitated with normal rabbit IgG or antibodies against H4K16Ac and H4. Precipitated genomic DNA was analyzed through real-time PCR using different primers for the different regions of PAI-1 promoter. The β-actin promoter (−204 to −59 bp) was used as a negative control. Data are shown as the mean±SEM of three independent experiments. **P < 0.01 and ***P < 0.001 vs. Ad-GFP. (G) SIRT1-H363Y overexpression decreased H4K16Ac level on the PAI-1 promoter to a much lesser degree compared with that of SIRT1-WT overexpression. Senescent HUVECs were infected with Ad-GFP, Ad-SIRT1, or Ad-SIRT1 H363Y and cultured for an additional 48 h. The chromatin was immunoprecipitated with normal rabbit IgG or antibodies against H4K16Ac and H4. Precipitated genomic DNA was analyzed through real-time PCR using different primers for the different regions of PAI-1 promoter. The β-actin promoter (−204 to −59 bp) was used as a negative control. Data are shown as the mean±SEM of three independent experiments. *P < 0.05, **P < 0.01 and ***P < 0.001 vs. Ad-SIRT1.

As a histone deacetylase, SIRT1 negatively regulates p66Shc expression through epigenetic chromatin modification, and as demonstrated in our previous study (Zhou *et al*., 2011), we examined whether SIRT1 regulates PAI-1 transcription at the chromatin level. First, we used ChIP assays to determine whether SIRT1 binds to the PAI-1 promoter in HUVECs. Consistent with our previous study, SIRT1 displayed strong binding to the p66Shc promoter and did not bind to the β-actin promoter. Importantly, SIRT1 also specifically bound to the PAI-1 promoter (−572 to −454 bp) (Fig. 6A,B). However, EX527 treatment markedly inhibited SIRT1 binding to the PAI-1 promoter (Fig. 6C). To examine whether SIRT1 affects the binding of acetylated histones on the PAI-1 promoter, the levels of two modified histone targets of SIRT1, H3K9Ac and H4K16Ac, were assayed on 1116 bp of the PAI-1 promoter from −1023 to +93 bp in young and senescent HUVECs. H3K9Ac level remained similar on different regions of the PAI-1 promoter and did not show significant difference compared with the negative control β-actin promoter in both young and senescent HUVECs (Fig. S8). However, H4K16Ac level was significantly higher on the region at −721 to −553 bp than on other regions (Fig. 6D) in young HUVECs. Moreover, higher H4K16Ac level was observed in senescent HUVECs compared with young HUVECs on the entire 1116-bp region of the PAI-1 promoter, particularly on the region at −721 to −553 bp (Fig. 6D). These findings suggest that H4K16Ac level, but not H3K9Ac, on the PAI-1 promoter plays a critical role in aging-induced PAI-1 mRNA overexpression. To further investigate whether SIRT1 regulates H4K16Ac level on the PAI-1 promoter, we treated young HUVECs with EX527 or overexpressed SIRT1 in senescent HUVECs. The results showed that EX527 treatment significantly increased H4K16Ac level on the entire region of the PAI-1 promoter, particularly on the region at −721 to −553 bp (Fig. 6E), while SIRT1 overexpression in senescent HUVECs almost completely removed H4K16Ac on the entire region of PAI-1 promoter, including the region at −721 to −553 bp (Fig. 6F). In addition, we also overexpressed a catalytically inactive SIRT1 mutant, SIRT1 H363Y, in senescent HUVECs, and found that SIRT1 H363Y overexpression also decreased H4K16Ac level, but to a much lesser degree compared with that of SIRT1 overexpression (Fig. 6G), suggesting that the deacetylase activity of SIRT1 is very important in the regulation of H4K16Ac level on the PAI-1 promoter. Furthermore, we detected the binding ability of Pol II on the PAI-1 promoter. Results showed that Pol II significantly bound to the PAI-1 promoter (Fig. S9A). And in senescent HUVECs, the binding level of Pol II on the PAI-1 promoter is similar to that in young HUVECs (Fig. S9B). These findings provide evidence that SIRT1 binds to the PAI-1 promoter, resulting in a decrease in the acetylation of histone H4K16 on the PAI-1 promoter region.

## Discussion

In the present study, we demonstrated for the first time to our knowledge that SIRT1 inhibition of PAI-1 expression protects against replicative senescence of endothelial cells, associated with vascular aging and the development of atherosclerosis. There are several major findings in this study. First, an internal negative correlation was detected between SIRT1 and PAI-1 expression in human atherosclerotic plaques and aortas of old mice, and the reduction of SIRT1 could induce the increased PAI-1 expression observed in senescent endothelial cells. Second, SIRT1 overexpression reversed the increased PAI-1 expression in senescent HUVECs and the aortas of old mice, accompanied by improved endothelial function and reduced arterial stiffness. Third, SIRT1-mediated inhibition of PAI-1 expression exhibited an antisenescence effect in HUVECs. Moreover, we demonstrated that SIRT1 is able to bind to the PAI-1 promoter, which epigenetically modifies the level of H4K16Ac on the PAI-1 promoter.

Endothelial senescence plays an important role in the development of atherosclerotic vascular diseases. Previous studies have shown that SIRT1 expression is reduced in the primary senescent porcine aortic endothelial cells and human atherosclerotic plaques (Menghini *et al*., 2009; Bai *et al*., 2012; Gorenne *et al*., 2013), and CDK5-mediated hyperphosphorylation of SIRT1 decreases its deacetylase activity and facilitates the development of endothelial senescence and atherosclerosis (Bai *et al*., 2012). Consistently, overexpression of SIRT1 in the endothelium protects against atherosclerotic plaque formation (Zhang *et al*., 2008). However, whether SIRT1 prevents replicative vascular senescence and by which SIRT1 facilitates the effects of antireplicative senescence and anti-atherogensis remain unclear. In the present study, we demonstrated an inverse correlation between SIRT1 and PAI-1 in human atherosclerotic plaques. We observed that SIRT1 overexpression significantly decreased the SA-β-gal-positive cell ratio in senescent HUVECs, indicating that SIRT1 represses HUVEC replicative senescence. We also observed that PAI-1 activation nearly blocked the antisenescence effect of SIRT1 overexpression in senescent HUVECs, while PAI-1 inhibition extinguished the pro-senescence effect of SIRT1 knockdown in young HUVECs, emphasizing the critical role of PAI-1 in the anti-aging effect of SIRT1 in endothelial replicative senescence. Moreover, both PAI-1 mRNA and protein expression were decreased in the aortas of SIRT1-Tg old mice compared with WT littermates, and SIRT1-Tg old mice showed improved endothelial function and decreased arterial stiffness. Furthermore, PAI-1 activity in mouse plasma was significantly decreased in the aortas of SIRT1-Tg old mice compared with those of WT mice. In addition, an exogenous stable form of PAI-1 CPAI, but not CPAI-Q123K, HPAI-RR, and PAI-L, blocked the antisenescence effect of SIRT1 *in vitro*, suggesting that the pro-senescence effect of PAI-1 is dependent on its antiproteolytic activity, and this result may explain the reduced plasma PAI-1 activity or aortic PAI-1 expression in SIRT1-Tg old mice contributes to the improved vascular function. Taken together, these data suggest that the repression of PAI-1 plays a crucial role in the anti-aging effect of SIRT1, leading to protection against vascular function decline and age-related cardiovascular diseases, such as atherosclerosis. Considering that other aging-related molecules, such as p53 and p21, play important roles in vascular aging (Kovacic *et al*., 2011), whereas deficiency of p53(Mercer *et al*., 2005) or p21(Khanna, 2009) has been reported to exacerbate atherosclerotic plaque formation in mice. We also detected the expression of these proteins in aortas from WT and SIRT1-Tg old mice and found that both mRNA and protein levels of p53 and p21 are decreased in aortas of SIRT1-Tg old mice (Fig. S5A,B). Additionally, other SIRT1 target proteins such as Notch and FOXOs (Guarani *et al*. 2011; Liu *et al*. 2012; Yao *et al*. 2012) play important roles in endothelial function. It will be interesting to further elucidate the involvement of these proteins in the regulation of replicative endothelial senescence and atherosclerosis by SIRT1.

Aging is associated with specific changes in chromatin. Senescent cells are characterized by the appearance of DNA damage, which can titrate out sirtuins and facilitate the accumulation of active chromatin markers, such as histone acetylation H3K9Ac and H4K16Ac and increased expression of pro-aging genes, which drive cell senescence (Rando & Chang, 2012). Previous studies have shown that SIRT1 affects the target gene promoter through histone deacetylation (Sun *et al*., 2007; Wang *et al*., 2008; Zhou *et al*., 2011), providing the possibility that SIRT1 maintains appropriate chromatin organization of the loci on promoters of some molecules sensitive to aging. In the present study, we observed that SIRT1 bound to the promoter of PAI-1, and this binding could be inhibited through EX527 treatment, suggesting that the deacetylase activity of SIRT1 is necessary for PAI-1 inhibition. Moreover, H4K16Ac, but not H3K9Ac, exhibited strong binding on the PAI-1 promoter, and H4K16Ac showed dramatically higher binding level on this region in senescent HUVECs than in young HUVECs, suggesting that H4K16Ac level on the PAI-1 promoter plays an important role in aging-induced PAI-1 upregulation. Furthermore, EX527 treatment significantly increased the level of H4K16Ac on the PAI-1 promoter, while SIRT1 overexpression in senescent HUVECs almost completely removed H4K16Ac on the PAI-1 promoter and SIRT1-H363Y, a catalytically inactive SIRT1 mutant, overexpression decreased H4K16Ac level to a much lesser degree compared with that of SIRT1-WT overexpression. Therefore, SIRT1 might inhibit PAI-1 upregulation in the aging process through epigenetic chromatin modification by reducing H4K16Ac level on the PAI-1 promoter. However, the exact role and detailed regulatory mechanism of H4K16Ac in endothelial senescence needs further investigation.

In conclusion, our data provide evidence that SIRT1 protects against replicative senescence in endothelial cells through a novel mechanism involving the epigenetic downregulation of PAI-1 expression. Identifying the direct molecular link between these two aging-related molecules might provide novel therapeutic opportunities for age-associated cardiovascular diseases, such as atherosclerosis.

## Experimental procedures

An expanded Experimental procedures section is available as supporting information in the online version of this article.

### Human atherosclerotic plaques and normal aortas

Human atherosclerotic plaques were obtained from 30 patients undergoing carotid endarterectomy, and normal carotid arteries were obtained from four patients after death. Atherosclerosis was excluded through morphological analysis, and all four patients were free of aortic diseases. The samples were obtained after informed consent and with approval of the institutional review boards (Chinese Academy of Medical Sciences and Peking Union Medical College Research Ethics Committee, Beijing, China).

### Cell culture and adenovirus generation

Endothelial cells were freshly isolated from human umbilical cord veins as previously described (Zhang *et al*., 2008). Human umbilical vein endothelial cells (HUVECs) were continuously passaged until the growth arrest status. The population doubling level (PDL) was calculated as previously described (Maciag *et al*., 1981). PDL6-8 HUVECs were used as young HUVECs, and PDL41-44 HUVECs were used as senescent HUVECs. Replication-defective adenoviral vectors expressing SIRT1 (Ad-SIRT1) or control green fluorescent protein (Ad-GFP) in addition to vectors for the adenovirus-mediated knockdown of SIRT1 (Ad-SIRT1 RNAi) or a control RNAi vector (Ad-U6) were generated using the AdEasy Vector Kit (Quantum Biotechnologies) as previously described (Zhang *et al*., 2008, 2010).

### Drugs

Sirtuin inhibitors sirtinol (cat. #S7942), nicotinamide (cat. #N3376), suramin (cat. #S2671), and EX527 (cat. #E7034) were purchased from Sigma-Aldrich Co., LLC. St. Louis, USA. The SIRT1 specific activator SRT1720 was from Selleck chemicals, Houston, TX. Human PAI-1 wild-type latent fraction (cat. #PAI-L), human PAI-1 stable mutant form (cat. #CPAI), human PAI-1 stable vitronectin reduced binding mutant (cat. #CPAI-Q123K), and human PAI-1 substrate form –P12 arginine P14 arginine double mutant (cat. #HPAI-RR) were purchased from Molecular Innovations, Inc., Novi, MI, USA. PAI-1 inhibitor PAI-039 (Tiplaxtinin, cat. #1383) was purchased from Axon Medchem BV, Groningen, the Netherlands.

### Animals

Male endothelium-specific SIRT1-Tg mice as previously described (Zhang *et al*., 2008), and WT littermates at 2–3 months old were used as young mice, and 20- to 22-month-old mice were used as old mice. 20- to 22-month-old SIRT1(+/+) and SIRT1(+/−) mice were used as old mice (McBurney *et al*., 2003). All animal experiments were performed in accordance with the Guide for the Care and Use of Laboratory Animals published by the US National Institutes of Health (NIH Publication No. 85-23, revised 1996).

### Senescence-associated β-galactosidase staining

Senescence-associated β-galactosidase (SA-β-gal) activity was determined as previously described (Dimri *et al*., 1995). HUVECs were counterstained with 2.5 μg mL^-1^ DAPI, and the proportion of SA-β-gal activity-positive cells was quantified using light and fluorescence microscopy.

### Isometric tension studies

The thoracic aortas were removed and placed in cold Krebs solution, cleaned of fat and loose connective tissue, and sectioned into 4-mm-long rings. The sectioned rings were mounted on stainless steel hooks and suspended in 10-mL tissue baths. The bath was filled with Krebs solution (at 37°C, pH 7.4, with 95% O_2_/5% CO_2_) of the following compositions (mm): NaCl 120, KCl 5.5, MgCl_2_∙6H_2_O 1.2, NaH_2_PO_4_ 1.2, CaCl_2_ 2.5, NaHCO_3_ 20, and glucose 10. The preload was 0.75 g, and the rings were equilibrated for 60 min. The Krebs buffer solution in the tissue bath was replaced every 15 min. At the end of the equilibration period, the maximal force generated by addition of 3 × 10^−6^ m phenylephrine (Sigma-Aldrich) was determined. To evaluate endothelium-dependent relaxations, the rings were preconstricted with phenylephrine (3 × 10^−6^ m) to obtain a stable plateau and then a cumulative dose–response curve to acetylcholine (10^−9^ to 10^−4^ m; Sigma-Aldrich) was obtained. The NO-donor sodium nitroprusside (10^−10^ to 10^−5^ m; Sigma-Aldrich) was added to examine endothelium-independent relaxations.

### Transit-time method for performing PWV measurements

The transit-time method estimates the pulse wave velocity (PWV) from the pressure wave transit time between two measurement locations separated by a known distance as previously described (Williams *et al*., 2007). Transit-time measurements were performed using a Visual Sonics Vevo770 ultrasound biomicroscope (Visual Sonics Inc., Toronto, ON, Canada). Results were processed using visual sonics analysis software.

### ChIP

Chromatin immunoprecipitation (ChIP) assays were performed in HUVECs as previously described (Wang *et al*., 2008). Primer sequences for specific genes are presented Table S1 (Supporting information).

### Statistics

Data analysis was performed using spss version 13.0 (SPSS, Inc., Chicago, IL, USA). Data are presented as the means ± standard error of mean (SEM). Paired data were compared using Students’ t-tests. Differences among groups were determined with 1-way or 2-way analysis of variance (ANOVA) with repeated measures, followed by Bonferroni post hoc test. A probability value of 0.05 was considered significant.

## Supporting Information

**Fig. S1** SIRT1, PAI-1, and p21 protein expressions in 30 AS plaques.

**Fig. S2** SA-β-gal staining (A) and Hoechest staining (B) on young and senescent HUVECs.

**Fig. S3** Analysis of p16 expression in HUVECs (A) and assay of plasma PAI-1 level of mice (B).

**Fig. S4** PAI-1 expression in 293A treated with sirtuin inhibitors and in HUVECs treated with drugs as indicated.

**Fig. S5** P53 and p21 mRNA and protein expression in the aortas of old WT and SIRT1-Tg mice.

**Fig. S6** SIRT1 and PAI-1 protein expressions in the aortas of old SIRT1(+/+) and SIRT1(+/-) mice.

**Fig. S7** The PAI-1 promoter activity affected by SIRT1 overexpression or SIRT1 inhibitor using the dual-luciferase reporter assay.

**Fig. S8** H3K9Ac level on the PAI-1 promoter by ChIP assays performed with chromatin prepared from young and senescent HUVECs.

**Fig. S9** Pol II level on the PAI-1 promoter by ChIP assays performed with chromatin prepared from young and senescent HUVECs.

**Table S1** Primer sequences used in the article.
