# Supplementary figures and images for: SIRT1-mediated epigenetic downregulation of plasminogen activator inhibitor-1 prevents vascular endothelial replicative senescence
Source: Aging Cell. 2014 Jul 18;13(5):890–9. doi: 10.1111/acel.12247 (PMC4331759; doi:10.1111/acel.12247)

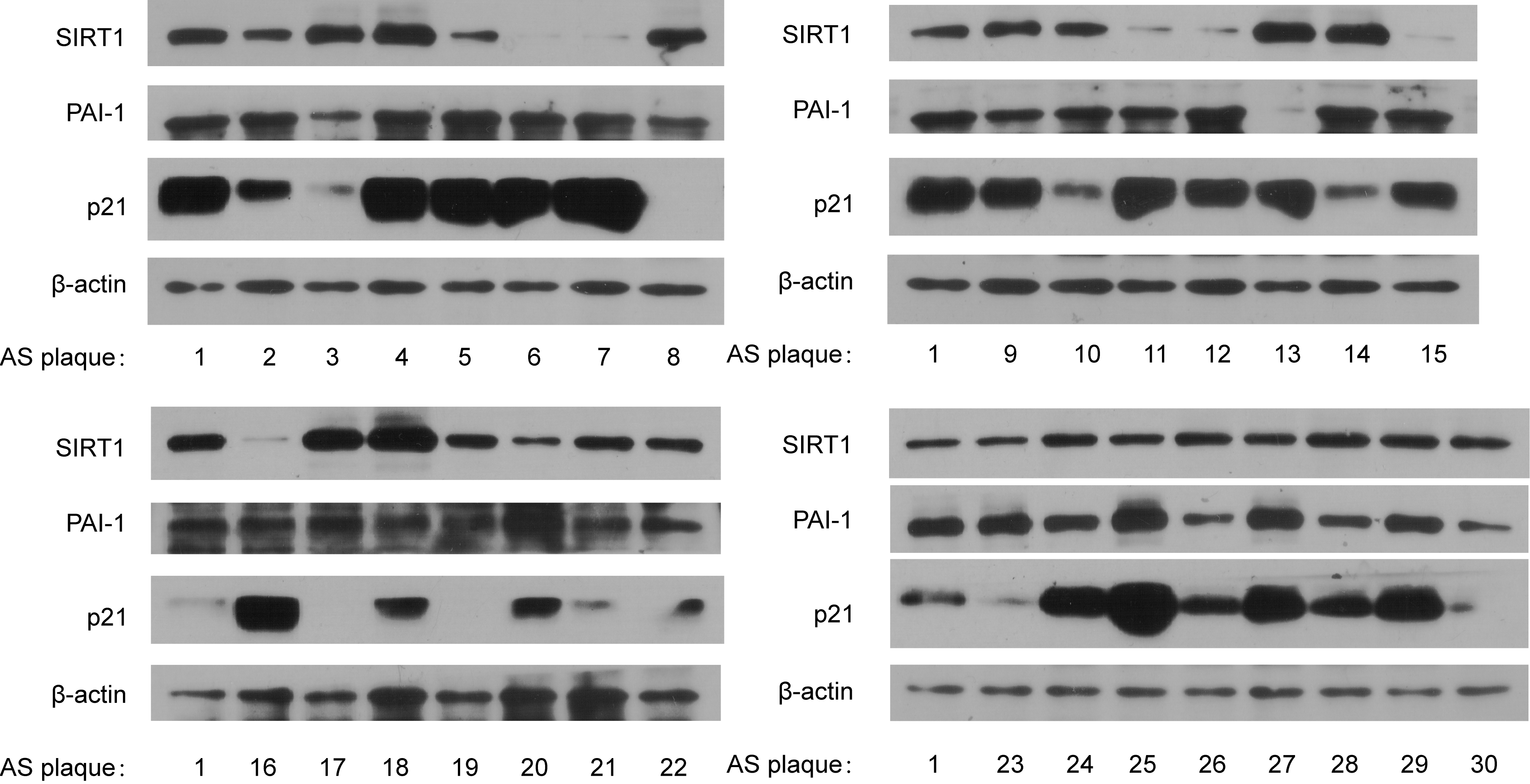

Supplement: Supplementary file 1 — Fig. S1 SIRT1, PAI-1, and p21 protein expressions in 30 AS plaques. [file acel0013-0890-sd1.tif]

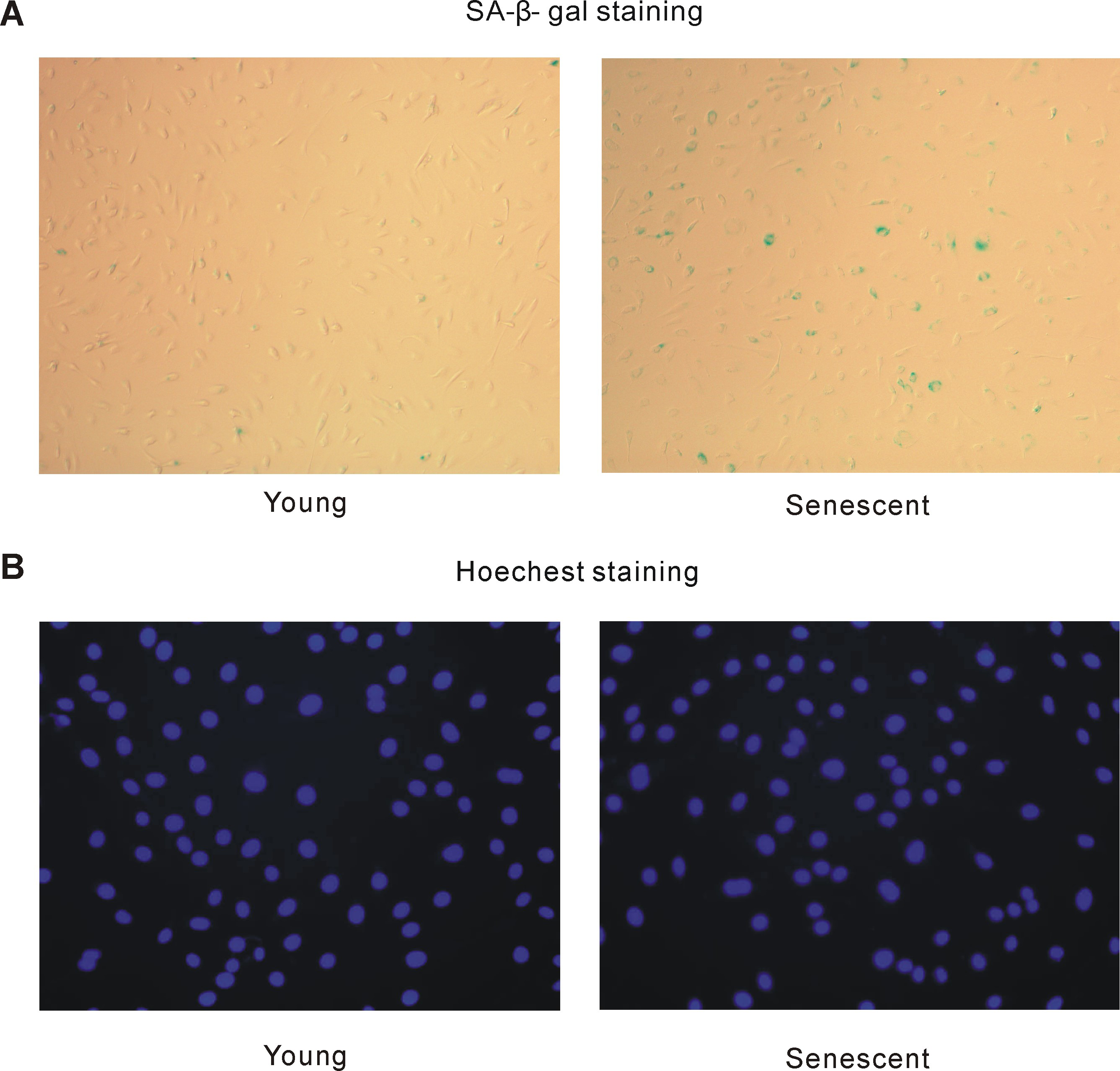

Supplement: Supplementary file 2 — Fig. S2 SA-β-gal staining (A) and Hoechest staining (B) on young and senescent HUVECs. [file acel0013-0890-sd2.tif]

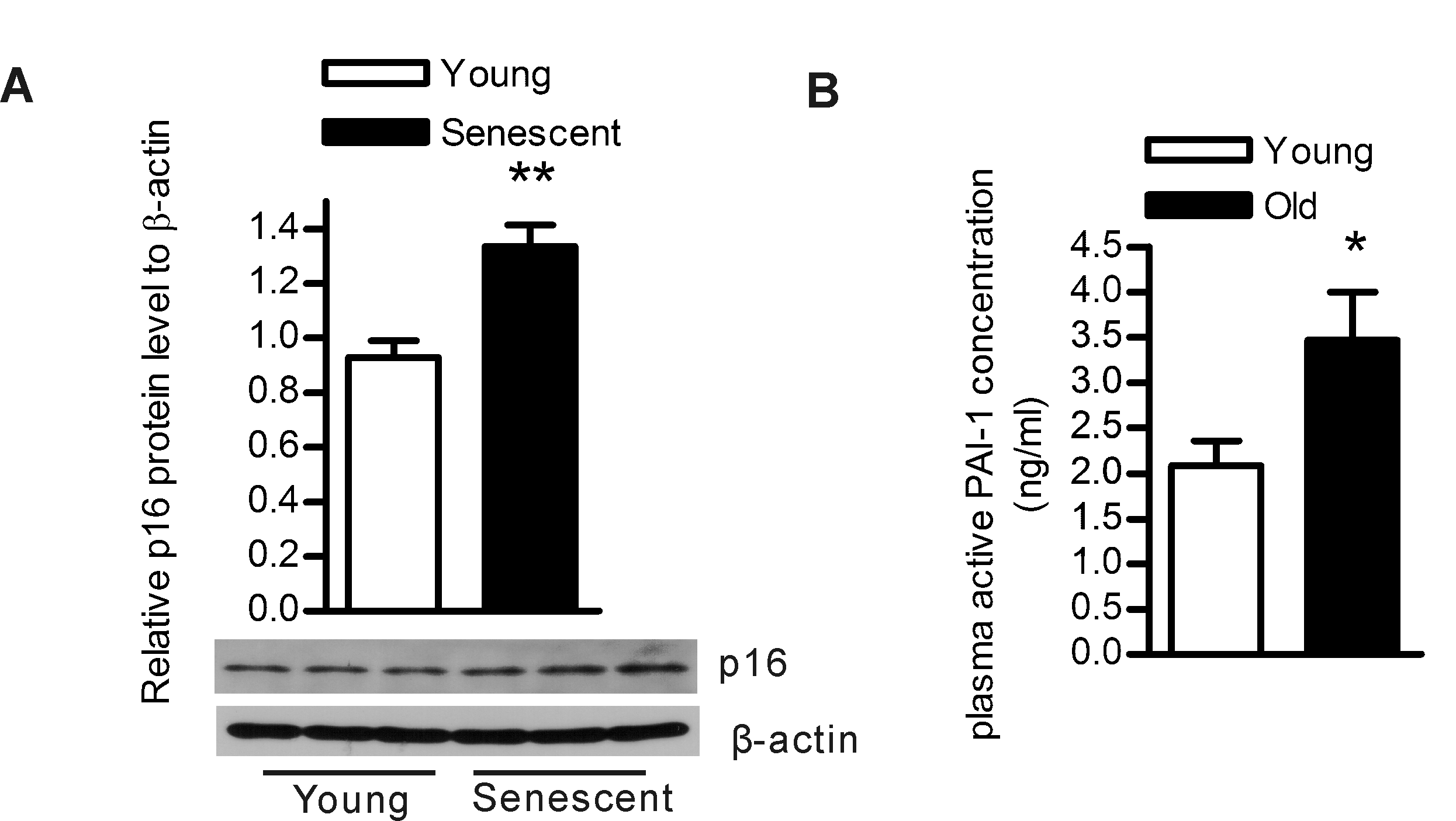

Supplement: Supplementary file 3 — Fig. S3 Analysis of p16 expression in HUVECs (A) and assay of plasma PAI-1 level of mice (B). [file acel0013-0890-sd3.tif]

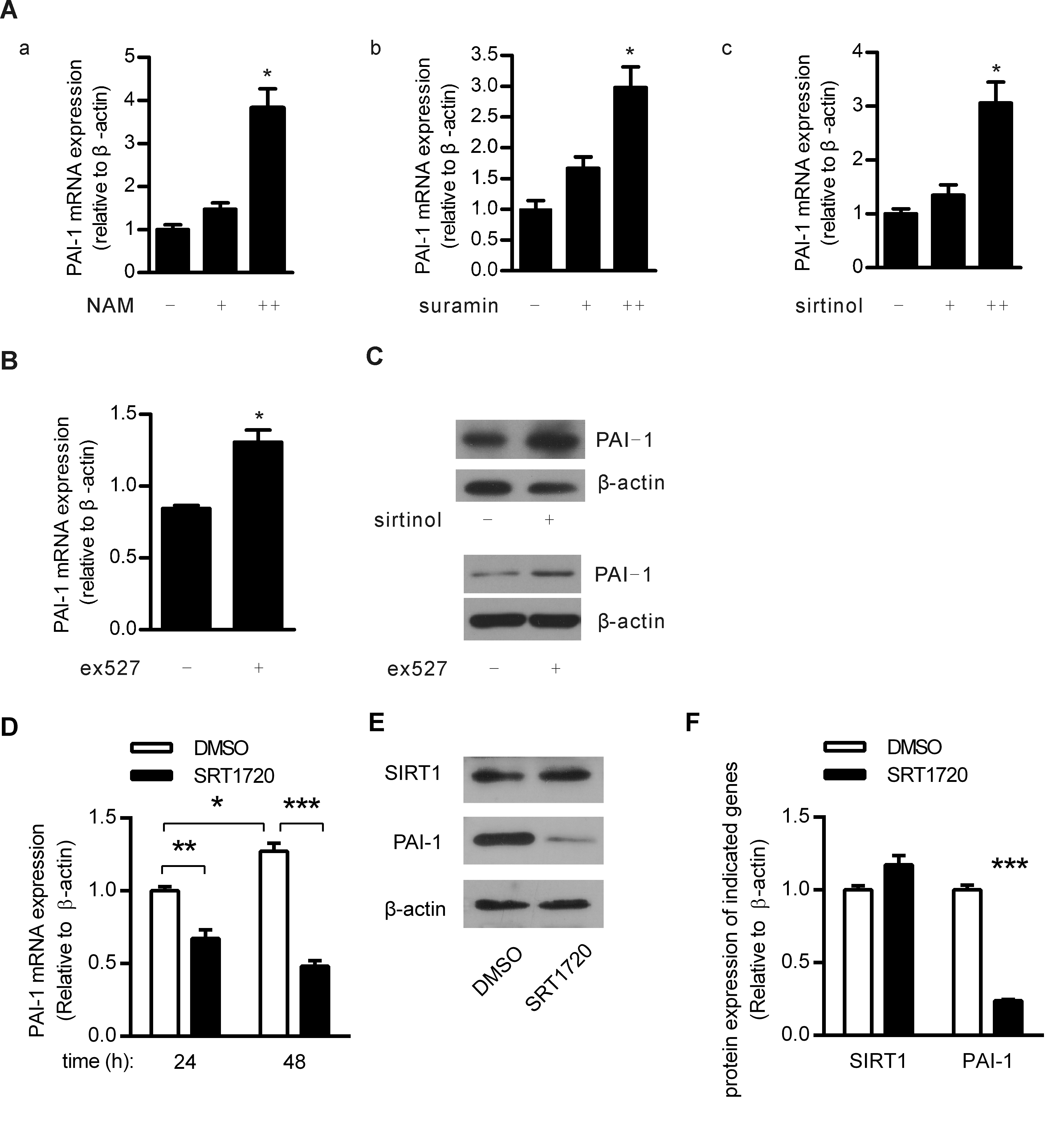

Supplement: Supplementary file 4 — Fig. S4 PAI-1 expression in 293A treated with sirtuin inhibitors and in HUVECs treated with drugs as indicated. [file acel0013-0890-sd4.tif]

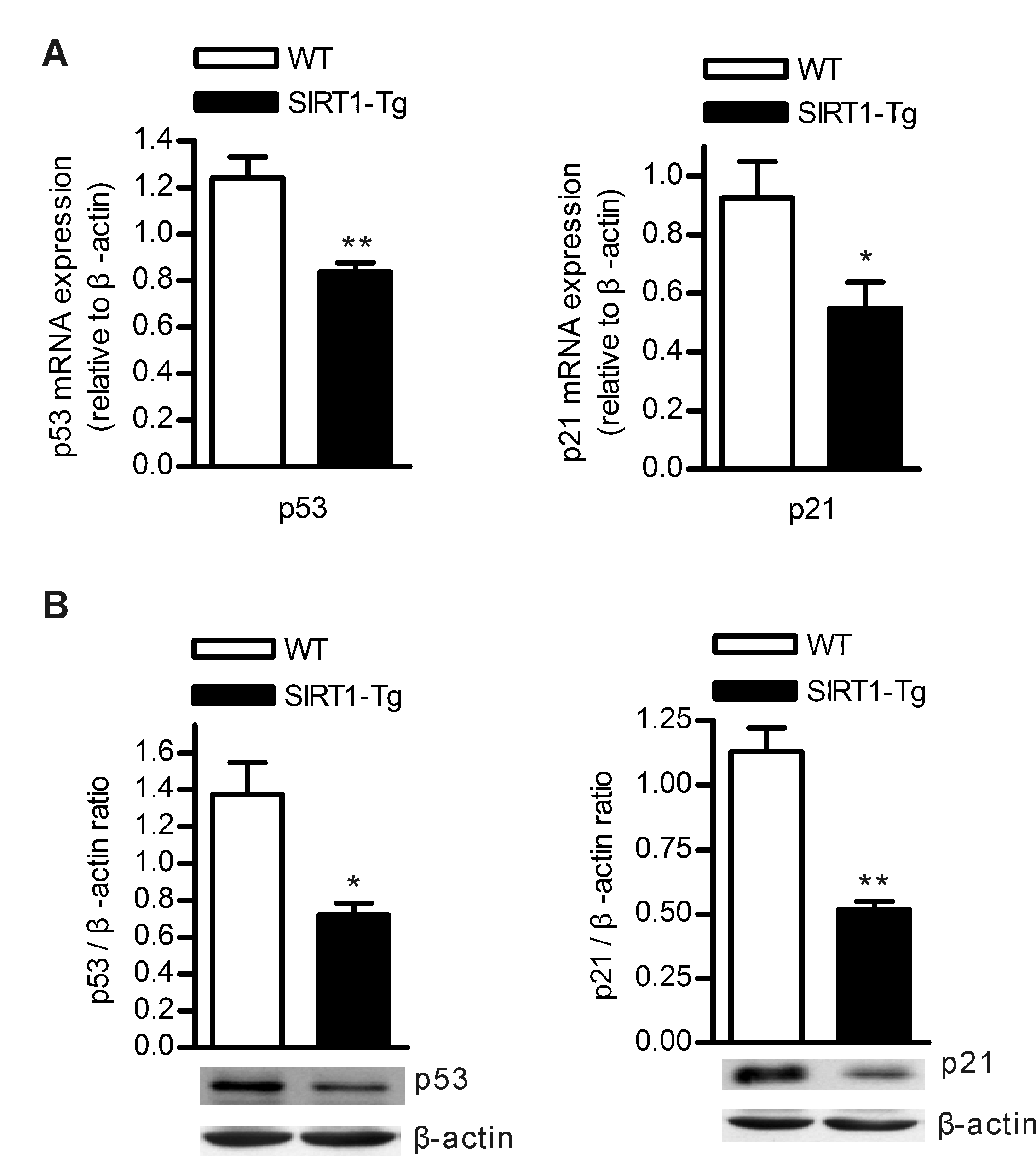

Supplement: Supplementary file 5 — Fig. S5 P53 and p21 mRNA and protein expression in the aortas of old WT and SIRT1-Tg mice. [file acel0013-0890-sd5.tif]

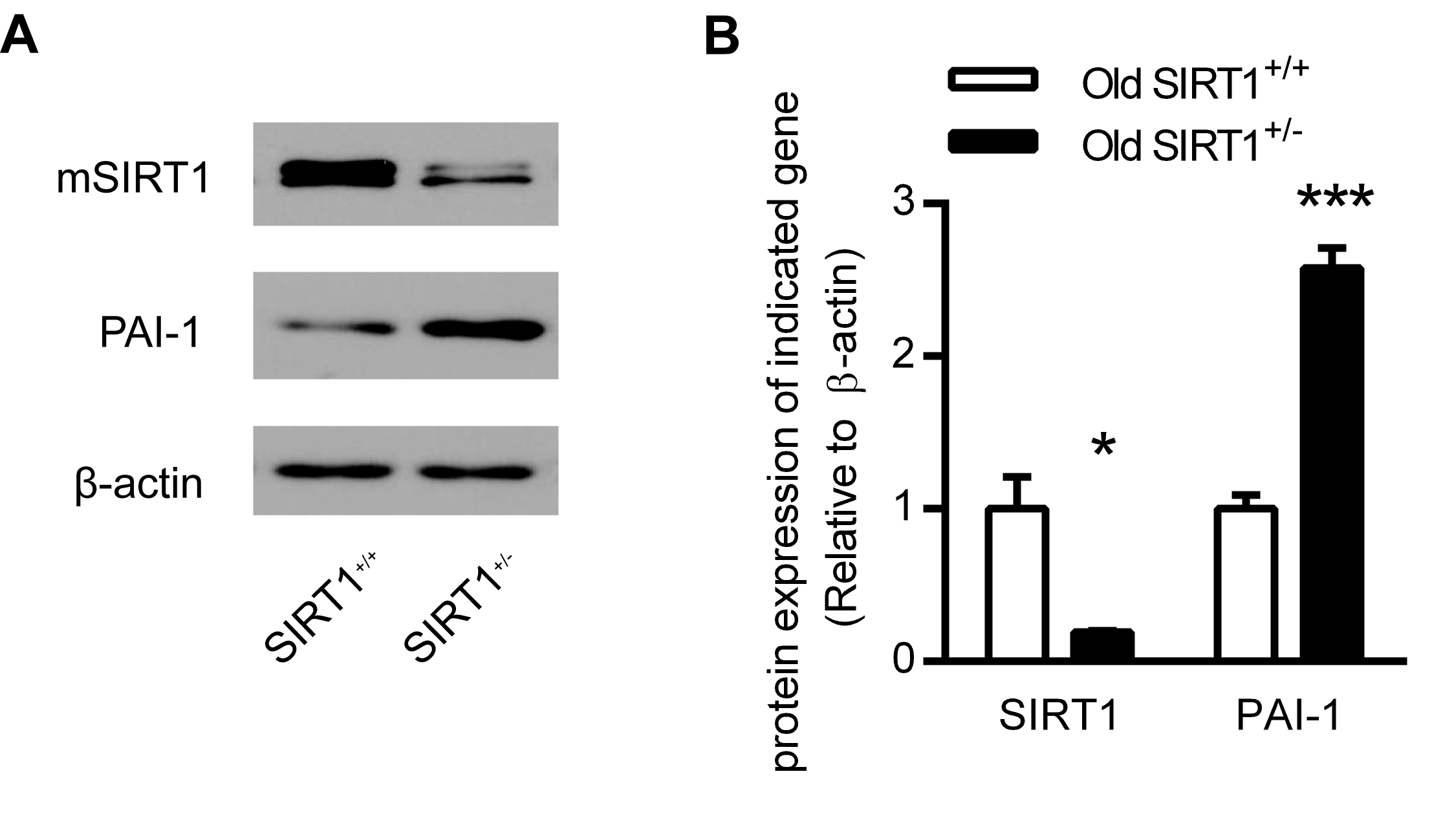

Supplement: Supplementary file 6 — Fig. S6 SIRT1 and PAI-1 protein expressions in the aortas of old SIRT1(+/+) and SIRT1(+/-) mice. [file acel0013-0890-sd6.tif]

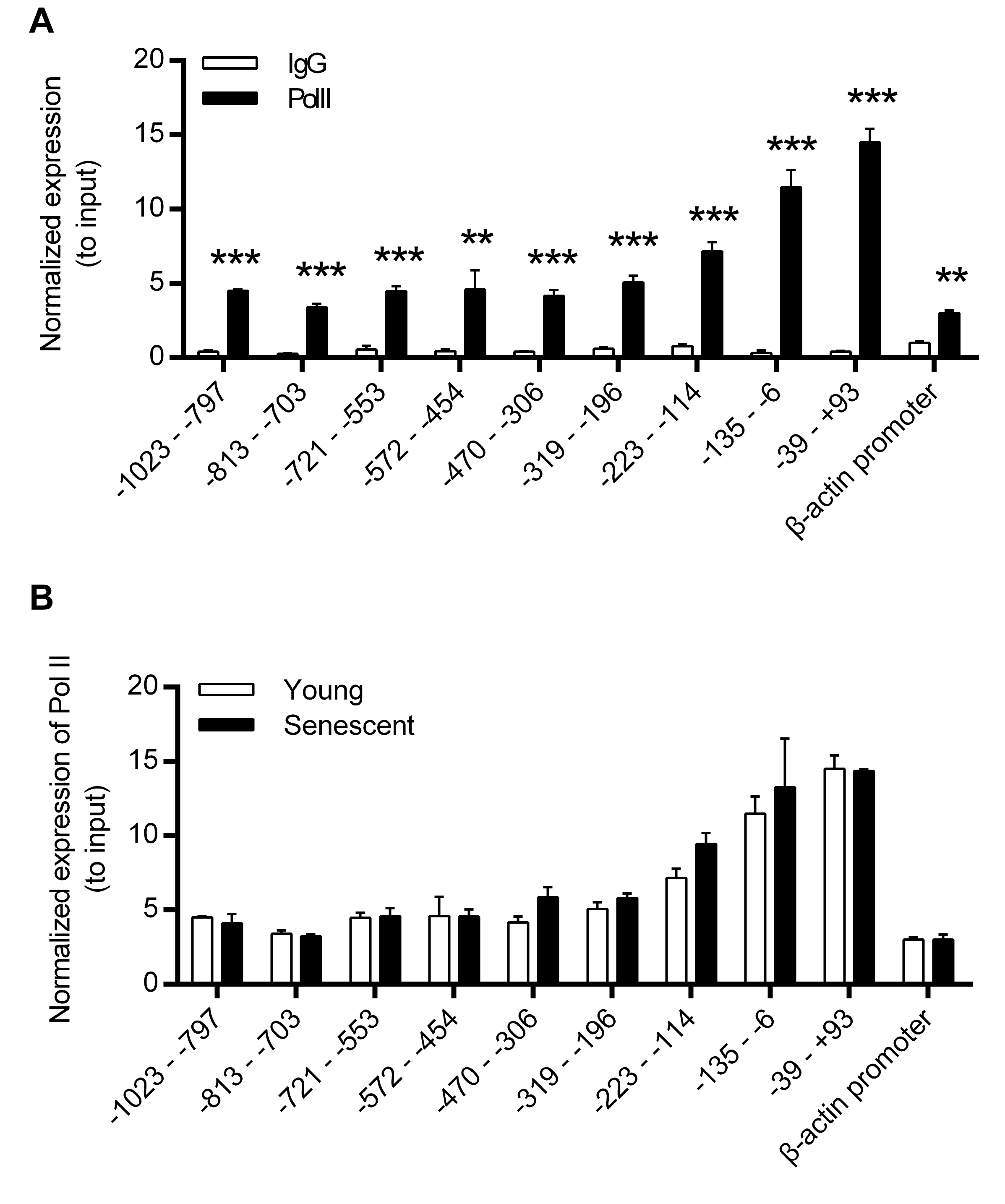

Supplement: Supplementary file 9 — Fig. S9 Pol II level on the PAI-1 promoter by ChIP assays performed with chromatin prepared from young and senescent HUVECs. [file acel0013-0890-sd9.tif]
