# Supplementary material for: SIRT1-mediated epigenetic downregulation of plasminogen activator inhibitor-1 prevents vascular endothelial replicative senescence
Source: Aging Cell. 2014 Jul 18;13(5):890–9. doi: 10.1111/acel.12247 (PMC4331759; doi:10.1111/acel.12247)
Supplement: Supplementary file 10 — Table S1 Primer sequences used in the article. [file acel0013-0890-sd10.doc]

**Table S1**

| **Gene** | **Accession** |  | **Sequences (5’-3’)** | **Length** | **Amplicon** |
| --- | --- | --- | --- | --- | --- |
| Human β-actin | NM_001101 | for | TCGTGCGTGACATTAAGGAG | 20bp | 245bp |
|  |  | rev | GATGTCCACGTCACACTTCA | 20bp |  |
| Human SIRT1 | NM_012238 | for | GATTGGCACAGATCCTCGAA | 20bp | 280bp |
|  |  | rev | GTCTACAGCAAGGCGAGCATA | 21bp |  |
| Human PAI-1 | NM_000602 | for | AAGGCACCTCTGAGAACTTCA | 21bp | 61bp |
|  |  | rev | CCCAGGACTAGGCAGGTG | 18bp |  |
| Human p21 | NM_078467 | for | GCAGACCAGCATGACAGATTT | 21bp | 70bp |
|  |  | rev | GGATTAGGGCTTCCTCTTGGA | 21bp |  |
| Mouse β-actin | NM_007393 | for | CCTTCCTTCTTGGGTATGGAATC | 23bp | 107bp |
|  |  | rev | AGCACTGTGTTGGCATAGAGGT | 22bp |  |
| Mouse SIRT1 | NM_019812 | for | GATTGGCACAGATCCTCGAA | 20bp | 280bp |
|  |  | rev | GTCTACAGCAAGGCGAGCATA | 21bp |  |
| Mouse PAI-1 | NM_008871 | for | CTCCGAGAATCCCACACAG | 19bp | 192bp |
|  |  | rev | ACTTTGAATCCCATAGCATC | 20bp |  |
| Mouse p53 | NM_011640 | for | CCGGGTGGAAGGAAATTTGT | 20bp | 89bp |
|  |  | rev | CCTCGGGTGGCTCATAAGGTA | 21bp |  |
| Mouse p21 | NM_007669 | for | TCTCAGGGCCGAAAACGGAG | 20bp | 169bp |
|  |  | rev | ACACAGAGTGAGGGCTAAGG | 20bp |  |
| Human PAI-1 promoter (-1023~-797) | NM_000602 | for | GAGGACCACTGCTCCACAGAAT | 22bp | 227bp |
|  |  | rev | AAGCTTGGTAAGGAAACAGGAGA | 23bp |  |
| Human PAI-1 promoter (-813~-703) | NM_000602 | for | GTTTCCTTACCAAGCTTTTACCAT | 24bp | 111bp |
|  |  | rev | GGCTCTCTTGTGTCAACAACCTT | 23bp |  |
| Human PAI-1 promoter (-721~-553) | NM_000602 | for | TTGTTGACACAAGAGAGCCCTC | 22bp | 169bp |
|  |  | rev | CAGCCACGTGATTGTCTAGGTT | 22bp |  |
| Human PAI-1 promoter (-572~-454) | NM_000602 | for | CCTAGACAATCACGTGGCTGGCT | 23bp | 119bp |
|  |  | rev | TTTCCCCCAGGGCTGTCCA | 19bp |  |
| Human PAI-1 promoter (-470~-306) | NM_000602 | for | GACAGCCCTGGGGGAAAACTT | 21bp | 165bp |
|  |  | rev | TGTCTGCCATGCCGGGTGA | 19bp |  |
| Human PAI-1 promoter (-319~-196) | NM_000602 | for | CGGCATGGCAGACAGTCAA | 19bp | 124bp |
|  |  | rev | AGCATTCAGGAACAATTGAGCA | 22bp |  |
| Human PAI-1 promoter (-223~-114) | NM_000602 | for | GGGGTTTGCTCAATTGTTCCT | 21bp | 110bp |
|  |  | rev | CCCTCGACACCTCCCTCTCT | 20bp |  |
| Human PAI-1 promoter (-135~-6) | NM_000602 | for | CCAGAGAGGGAGGTGTCGAG | 20bp | 130bp |
|  |  | rev | GGCCACTGCCTCCTTTTATAC | 21bp |  |
| Human PAI-1 promoter (-39~+93) | NM_000602 | for | CTGCCCACATCTGGTATAAAAGG | 23bp | 132bp |
|  |  | rev | CTGCTGAGCTGCAGGAATTCA | 21bp |  |
| Human p66Shc promoter (-508~-250) | NM_003029 | for | CTTACTGTATGGGGTAGCGGTT | 22bp | 259bp |
|  |  | rev | ACGGAAAGGAAGGAGATAGGAG | 22bp |  |
| Human β-actin promoter (-204~-59) | NM_001101 | for | TCCTCCTCTTCCTCAATCTCG | 21bp | 146bp |
|  |  | rev | AAGGCAACTTTCGGAACGG | 19bp |  |
